# Machine learning-based personalized risk prediction model for breast cancer-related lymphedema after surgery

**DOI:** 10.3389/fonc.2025.1729340

**Published:** 2025-12-04

**Authors:** Xuemei Peng, Yanfang Ai, Weiying Xu, Jingling Hong, Qinyan Li, Jianfen Liu

**Affiliations:** Department of Breast Surgery, Jiangxi Cancer Hospital & Institute, The Second Affiliated Hospital of Nanchang Medical College, Nanchang, Jiangxi, China

**Keywords:** breast cancer, lymphedema, machine learning, prediction model, cancer

## Abstract

**Objective:**

Breast cancer-related lymphedema (BCRL) is a common postoperative complication that significantly impairs patients’ quality of life. This study aims to develop a machine learning-based personalized risk prediction model for BCRL by integrating multimodal clinical and behavioral data, thereby providing scientific support for early identification and intervention in high-risk individuals.

**Methods:**

Clinical and follow-up data were collected from patients who underwent breast cancer surgery between June 2020 and June 2025. A total of 38 variables were analyzed using the Least Absolute Shrinkage and Selection Operator (LASSO) method for feature selection. Nine machine learning algorithms were developed, and their performance was evaluated using metrics including accuracy, sensitivity, specificity, positive predictive value, negative predictive value, F1-score, and the area under the receiver operating characteristic curve (AUC). The optimal model was further interpreted using Shapley Additive Explanations (SHAP) to enable individualized risk assessment.

**Results:**

A total of 368 eligible patients were included and randomly divided into a training set (n = 257) and a validation set (n = 111) at a 7:3 ratio. Among them, 98 patients (26.63%) developed BCRL. LASSO regression identified 12 features most predictive of BCRL. Among all models, logistic regression demonstrated the best performance, with an AUC of 0.937, accuracy of 0.793, sensitivity of 0.937, and specificity of 0.740 in the validation set. BMI, lymph node dissection level, and lymph node status were identified as the most influential predictors contributing to model performance.

**Conclusion:**

A logistic regression model, combined with SHAP-based interpretation, enables personalized risk prediction of BCRL in postoperative breast cancer patients. This approach may provide robust support for clinical risk stratification and intervention planning.

## Background

Breast cancer (BC) is one of the most common malignancies among women worldwide, with an incidence that continues to rise. Recent studies indicate that advancements in screening technologies and comprehensive treatment strategies have significantly improved survival rates in breast cancer patients (1).However, increasing attention is being paid to postoperative complications.

Breast cancer-related lymphedema (BCRL) is among the most prevalent chronic complications following breast cancer surgery, affecting approximately one-fifth of patients (2). BCRL primarily results from impaired lymphatic drainage, leading to the accumulation of protein-rich fluid in the interstitial space and the development of secondary lymphedema (3, 4). Clinically, patients often present with upper limb swelling, heaviness, pain, progressive fibrosis, and skin hardening (5).

Numerous studies have identified several risk factors associated with BCRL, including axillary lymph node dissection (ALND), adjuvant radiotherapy and chemotherapy, tumor staging, obesity, and age (6, 7). Although these findings have provided valuable insights into the pathophysiology of BCRL, there remains a lack of comprehensive predictive tools that integrate multiple variables to assess individual risk.

In recent years, advanced technologies such as machine learning (ML) have emerged as powerful tools for modeling cancer treatment outcomes and prognostic parameters (8). Compared to traditional statistical approaches, ML methods are better suited for handling high-dimensional data with multiple predictors, offering improved accuracy in personalized risk prediction and demonstrating strong potential for clinical application (9).

This study aims to construct multiple ML-based models to predict the individualized risk of developing BCRL after surgery. In addition, the interpretability of the models is enhanced using Shapley Additive Explanations (SHAP), which enables clinicians to identify key risk factors more clearly, thereby supporting postoperative management and intervention strategies to reduce the incidence of BCRL.

## Methods

### Study population

Female patients diagnosed with unilateral breast cancer who underwent either unilateral radical mastectomy or modified radical mastectomy between June 2020 and June 2025 were recruited for this study. The inclusion criteria were as follows: (1) pathologically confirmed breast cancer with surgical treatment; (2) female sex; (3) age ≥18 years; (4) normal reading and comprehension abilities without cognitive or communication disorders; and (5) provision of written informed consent.

Patients were excluded if they met any of the following criteria: (1) bilateral breast cancer; (2) malignancies at non-breast sites; (3) history of tumor recurrence or metastasis; (4) prior diagnosis of lymphedema or other lymphatic system disorders; (5) inability or unwillingness to participate, or incomplete clinical records; (6) presence of serious comorbidities such as severe cardiac, cardiogenic, or renal failure; (7) congenital or acquired upper limb abnormalities (e.g., deformity, amputation, tumor, or scarring) that prevented arm circumference measurement; or (8) inability of the patient or caregiver to complete the questionnaire using a smartphone or WeChat.

### Study variables

A data collection form comprising five sections and a total of 38 variables was used (**Table 1**). The first four sections—demographic, tumor characteristics, surgical and treatment-related factors, and postoperative status—were extracted from the electronic medical record system. The fifth section, containing behavioral data relevant to reducing lymphedema risk, was collected using a structured questionnaire administered via WeChat (a messaging application), telephone interviews, or outpatient follow-ups. After comprehensive explanation of the study’s purpose and significance, the questionnaire was completed independently by participants or their legal guardians/next of kin with informed consent.

**Table 1:** The variables in this study and their corresponding categories used for predicting BCRL.

Category	Variables
Demographics	Age, BMI, dominant side affected, hypertension, diabetes, menopause status
Tumor characteristics	Path type, clinical stage, T stage, N stage, M stage, ER detection, PR detection, HER2 detection, the number of lymph node positives, tumor location, tumor side
Surgical and treatment-related factors	Breast surgical approach, level of axillary lymph node dissection (ALND), the number of lymph nodes removed, lymph node surgical approach, type of surgical incision, neoadjuvant chemotherapy, adjuvant chemotherapy, postoperative radiotherapy, endocrine therapy, whether the limb used for blood draws and injections is the same as the surgical limb, whether the limb used for blood pressure measurement is the same as the surgical limb
Postoperative outcomes	Postoperative complications, recurrence of the tumor
Behavioral and lifestyle factors	Exercise time, exercise frequency, exercise type, healthy Diet, education BCRL, avoid heavy lifting, skin clean dry, rest f pain

To enhance the reliability and validity of self-reported behavioral variables, we developed a structured questionnaire to collect postoperative behavior-related data, including exercise frequency, avoidance of heavy lifting, and adherence to lymphedema prevention recommendations. The questionnaire items were formulated based on a systematic review of previously published studies and relevant clinical guidelines for reducing the risk of breast cancer-related lymphedema (10–12).

The preliminary version of the questionnaire was reviewed by a multidisciplinary expert panel consisting of two breast surgeons, two oncology nurses, and one rehabilitation specialist to guarantee its content validity and clarity. Subsequently, a pilot test involving 20 postoperative breast cancer survivors was conducted to evaluate the comprehensibility of the items and the overall structural coherence. Revisions were made to the wording and logical flow based on participant feedback. Furthermore, the internal consistency of the multi-item behavioral scales was assessed using Cronbach’s α coefficient, which exceeded 0.70, indicating an acceptable level of reliability for measuring behavioral constructs.

### Assessment of lymphedema

The diagnostic criterion for BCRL was based on objective circumference measurements. A non-elastic measuring tape was used to measure the circumference at four standardized points on both upper limbs: the wrist crease, 10 cm above the wrist crease, the antecubital fossa, and 10 cm above the antecubital fossa. A difference of ≥2.0 cm between the affected and unaffected arms at any site was defined as BCRL (13). The occurrence of BCRL was set as the follow-up outcome event, and follow-up ended in June 2025.

### Model development and evaluation

Feature selection was performed using the Least Absolute Shrinkage and Selection Operator (LASSO) regression via R software (glmnet v4.1.2), which adjusts for model complexity and variable selection. After identifying relevant predictors from the full set of independent variables, patients were randomly divided into a training set (n = 257) and a validation set (n = 111) in a 7:3 ratio.

Multiple machine learning classification models were constructed to analyze and compare the importance of variables across both datasets. The best-performing model was selected based on these comparisons. The area under the receiver operating characteristic (ROC) curve (AUC) was used to assess the discriminative ability of each model. Additional evaluations included decision curve analysis (DCA), calibration plots, and precision-recall (PR) curves. Shapley Additive Explanations (SHAP) were applied to provide both global model interpretability and individualized prediction insights.

### Clinical decision support framework

To facilitate the translation of our predictive model into clinical practice, we developed a structured decision support workflow. This framework outlines the systematic process from patient assessment to personalized intervention, comprising five key stages: Patient Assessment, Feature Extraction, Model Prediction, Risk Interpretation, Clinical Action.

### Statistical analysis

Comparisons of variables between the training and validation sets were conducted. Continuous variables were presented as means ± standard deviations and compared using the Mann–Whitney U test. Categorical variables were reported as frequencies and percentages, and compared using the chi-square test. A two-sided p-value < 0.05 was considered statistically significant. Data analysis was performed using SPSS version 28.0 and R version 4.4.3.

## Results

### Participant characteristics

A total of 368 postoperative breast cancer patients were enrolled in this study and categorized into the non-lymphedema group and the BCRL group, with 98 patients (26.63%) diagnosed with BCRL. Baseline characteristics between the two groups are summarized in **Table 2**. Significant differences were observed in variables including age, BMI, number of positive lymph nodes, number of dissected lymph nodes, hypertension status, pathological type, clinical stage, T stage, N stage, type of surgery, lymph node dissection level, type of lymph node surgery, neoadjuvant chemotherapy, adjuvant chemotherapy, postoperative radiotherapy, endocrine therapy, postoperative complications, frequency of postoperative exercise, BCRL-related health education, and avoidance of heavy lifting (p < 0.05). Additionally, baseline characteristics of the training and validation sets are presented in **Table 3**, with no statistically significant differences between the two sets (p > 0.05).

**Table 2:** Comparison of demographic, disease, and treatment information of postoperative breast cancer patients between different groups.

Variable	Non-lymphedema (n=270)	Lymphedema (n=98)	P value
Age, Mean (SD)	49.97 (4.78)	51.52 (5.58)	0.009
BMI, Mean (SD)	24.24 (2.43)	25.96 (2.21)	<0.001
Lymph Pos Num, Mean (SD)	1.64 (2.61)	3.91 (2.59)	<0.001
LN Removed, Mean (SD)	13.16 (5.26)	16.80 (4.25)	<0.001
Hypertension, N (%)			0.013
Yes	33 (12.2)	23 (23.5)	
No	237 (87.8)	75 (76.5)	
Diabetes, N (%)			0.296
Yes	17 (6.3)	10 (10.2)	
No	253 (93.7)	88 (89.8)	
Dominant Side Affected, N (%)			0.474
Right	130 (48.1)	52 (53.1)	
Left	140 (51.9)	46 (46.9)	
Menopause Status, N (%)			0.671
Premenopause	147 (54.4)	58 (59.2)	
Perimenopause	25 (9.3)	7 (7.1)	
Postmenopause	98 (36.3)	33 (33.7)	
Path Type, N (%)			0.036
Invasive	237 (87.8)	94 (95.9)	
Non-invasive	33 (12.2)	4 (4.1)	
Clinical Stage, N (%)			<0.001
Stage 0	3 (1.1)	0 (0.0)	
Stage I	97 (35.9)	8 (8.2)	
Stage II	155 (57.4)	60 (61.2)	
Stage III	12 (4.4)	29 (29.6)	
Stage IV	3 (1.1)	1 (1.0)	
T Stage, N (%)			<0.001
T0	3 (1.1)	1 (1.0)	
T1	114 (42.2)	12 (12.2)	
T2	141 (52.2)	71 (72.4)	
T3	11 (4.1)	13 (13.3)	
T4	1 (0.4)	1 (1.0)	
N Stage, N (%)			<0.001
N0	183 (67.8)	17 (17.3)	
N1	76 (28.1)	58 (59.2)	
N2	8 (3.0)	21 (21.4)	
N3	3 (1.1)	2 (2.0)	
M Stage=M1, N (%)	2 (0.7)	1 (1.0)	1.000
ER, N (%)			0.919
Positive	177 (65.6)	63 (64.3)	
Negative	93 (34.4)	35 (35.7)	
PR, N (%)			0.240
Positive	182 (67.4)	73 (74.5)	
Negative	88 (32.6)	25 (25.5)	
HER2, N (%)			0.634
Positive	50 (18.5)	21 (21.4)	
Negative	220 (81.5)	77 (78.6)	
Tumor Location, N (%)			0.782
Periareolar	31 (11.5)	7 (7.1)	
LIQ	20 (7.4)	7 (7.1)	
LOQ	49 (18.1)	18 (18.4)	
UIQ	49 (18.1)	21 (21.4)	
UOQ	121 (44.8)	45 (45.9)	
Tumor Side, N (%)			0.932
Right	129 (47.8)	48 (49.0)	
Left	141 (52.2)	50 (51.0)	
Surgery Type, N (%)			0.032
BCS	64 (23.7)	14 (14.3)	
Mastectomy	199 (73.7)	84 (85.7)	
Reconstruction	7 (2.6)	0 (0.0)	
ALND Level, N (%)			<0.001
None	119 (44.1)	18 (18.4)	
Level I	63 (23.3)	13 (13.3)	
Level II	49 (18.1)	10 (10.2)	
Level III	39 (14.4)	57 (58.2)	
LN Surgery Type, N (%)			<0.001
ALND	136(50.4)	86(87.8)	
SLNB	134 (49.6)	12 (12.2)	
Incision Type, N (%)			0.343
Crescent	18 (6.7)	10 (10.2)	
Laparoscopy	2 (0.7)	3 (3.1)	
Longitudinal	12 (4.4)	4 (4.1)	
Oblique	17 (6.3)	7 (7.1)	
Transverse	221 (81.9)	74 (75.5)	
Neoadjuvant chemotherapy, N (%)			<0.001
None	182 (67.4)	38 (38.8)	
Non-taxane	11 (4.1)	13 (13.3)	
Taxane	77 (28.5)	47 (48.0)	
Adjuvant chemotherapy, N (%)			0.018
None	98 (36.3)	24 (24.5)	
Non-taxane	35 (13.0)	8 (8.2)	
Taxane	137 (50.7)	66 (67.3)	
Radiotherapy, N (%)			<0.001
Yes	133 (49.3)	76 (77.6)	
No	137 (50.7)	22 (22.4)	
Endocrine Therapy, N (%)			0.003
Yes	162 (60.0)	76 (77.6)	
No	108 (40.0)	22 (22.4)	
Draw Inject Same Side, N (%)			0.484
Yes	137 (50.7)	45 (45.9)	
No	133 (49.3)	53 (54.1)	
BP Same Side, N (%)			0.654
Yes	137 (50.7)	53 (54.1)	
No	133 (49.3)	45 (45.9)	
Complication, N (%)			0.017
None	188 (69.6)	75 (76.5)	
Hematoma	1 (0.4)	4 (4.1)	
Infection	30 (11.1)	6 (6.1)	
Seroma	51 (18.9)	13 (13.3)	
Tumor Recur, N (%)			0.680
Yes	6 (2.2)	1 (1.0)	
No	264 (97.8)	97 (99.0)	
Exercise Time, N (%)			0.474
<30min	134 (51.9)	52 (46.9)	
≥30min	123 (48.1)	59 (53.1)	
Exercise Freq, N (%)			<0.001
<3/week	134(49.6)	80 (81.6)	
≥3/week	136 (50.4)	18 (18.4)	
Exercise Type, N (%)			0.674
Jog	90 (33.3)	37 (37.8)	
Walk	83 (30.7)	30 (30.6)	
Rehab	97 (35.9)	31 (31.6)	
Healthy Diet, N (%)			0.677
Yes	138 (51.1)	47 (48.0)	
No	132 (48.9)	51 (52.0)	
Edu BCRL, N (%)			<0.001
Yes	125 (46.3)	24 (24.5)	
No	145 (53.7)	74 (75.5)	
Avoid Heavy Lifting, N (%)			0.040
Yes	142 (52.6)	39 (39.8)	
No	128 (47.4)	59 (60.2)	
Skin Clean Dry, N (%)			0.666
Yes	141 (52.2)	48 (49.0)	
No	129 (47.8)	50 (51.0)	
Rest If Pain, N (%)			0.484
Yes	137 (50.7)	45 (45.9)	
No	133 (49.3)	53 (54.1)	

BMI, ​body mass index; ER, ​estrogen receptor; HER-2, ​human epidermal growth factor receptor-2; PR, ​progesterone receptor; LIQ, located in lower inner quadrant; LOQ, located in lower outer quadrant; UIQ, located in upper inner quadrant; UOQ, located in upper outer quadrant; BCS, breast-conserving surgery; ALND, ​axillary lymph node dissection; SLNB, ​sentinel lymph node biopsy; Draw Inject Same Side, whether blood draws/injections were performed on the surgical side; BP Same Side, whether blood pressure was measured on the surgical side; Skin Clean Dry, whether the skin is maintained clean and dry; Rest If Pain, whether appropriate rest is taken when experiencing pain.

**Table 3:** Baseline characteristics in training cohort and testing cohort.

Variable	Training Set (n = 257)	Testing Set (n =111)	P value
Age, Mean (SD)	50.40 (4.62)	51.15 (5.85)	0.064
BMI, Mean (SD)	24.62 (2.54)	24.88 (2.38)	0.352
Lymph Pos Num, Mean (SD)	2.29 (2.77)	2.14 (2.84)	0.630
LN Removed, Mean (SD)	14.21 (5.31)	13.95 (5.14)	0.674
Hypertension, N (%)			0.284
Yes	43 (16.7)	13 (11.7)	
No	214 (83.3)	98 (88.3)	
Diabetes, N (%)			0.474
Yes	21 (8.2)	6 (5.4)	
No	236 (91.8)	105 (94.6)	
Dominant Side Affected, N (%)			0.554
Right	124 (48.2)	58 (52.3)	
Left	133 (51.8)	53 (47.7)	
Menopause Status, N (%)			0.767
Premenopause	142 (55.3)	63 (56.8)	
Perimenopause	21 (8.2)	11 (9.9)	
Postmenopause	94 (36.6)	37 (33.3)	
Path Type, N (%)			1.000
Invasive	231 (89.9)	100 (90.1)	
Non-invasive	26 (10.1)	11 (9.9)	
Clinical Stage, N (%)			0.294
Stage 0	3 (1.2)	0 (0.0)	
Stage I	70 (27.2)	35 (31.5)	
Stage II	148 (57.6)	67 (60.4)	
Stage III	32 (12.5)	9 (8.1)	
Stage IV	4 (1.6)	0 (0.0)	
T Stage, N (%)			0.288
T0	4 (1.6)	0 (0.0)	
T1	81 (31.5)	45 (40.5)	
T2	153 (59.5)	59 (53.2)	
T3	17 (6.6)	7 (6.3)	
T4	2 (0.8)	0 (0.0)	
N Stage, N (%)			0.069
N0	136 (52.9)	64 (57.7)	
N1	92 (35.8)	42 (37.8)	
N2	26 (10.1)	3 (2.7)	
N3	3 (1.2)	2 (1.8)	
M Stage=M1, N (%)	3 (1.2)	0 (0.0)	0.557
ER, N (%)			0.791
Positive	166 (64.6)	74 (66.7)	
Negative	91 (35.4)	37 (33.3)	
PR, N (%)			0.727
Positive	180 (70.0)	75 (67.6)	
Negative	77 (30.0)	36 (32.4)	
HER2, N (%)			0.581
Positive	52 (20.2)	19 (17.1)	
Negative	205 (79.8)	92 (82.9)	
Tumor Location, N (%)			0.953
Periareolar	25 (9.7)	13 (11.7)	
LIQ	20 (7.8)	7 (6.3)	
LOQ	48 (18.7)	19 (17.1)	
UIQ	48 (18.7)	22 (19.8)	
UOQ	116 (45.1)	50 (45.0)	
Tumor Side, N (%)			0.511
Right	127 (49.4)	50 (45.0)	
Left	130 (50.6)	61 (55.0)	
Surgery Type, N (%)			0.680
BCS	56 (21.8)	22 (19.8)	
Mastectomy	195 (75.9)	88 (79.3)	
Reconstruction	6 (2.3)	1 (0.9)	
ALND Level, N (%)			0.708
None	91 (35.4)	46 (41.4)	
Level I	56 (21.8)	20 (18.0)	
Level II	42 (16.3)	17 (15.3)	
Level III	68 (26.5)	28 (25.2)	
LN Surgery Type, N (%)			0.915
ALND	156 (60.7)	66 (59.5)	
SLNB	101 (39.3)	45 (40.5)	
Incision Type, N (%)			0.231
Crescent	18 (7.0)	10 (9.0)	
Laparoscopy	3 (1.2)	2 (1.8)	
Longitudinal	15 (5.8)	1 (0.9)	
Oblique	15 (5.8)	9 (8.1)	
Transverse	206 (80.2)	89 (80.2)	
Neoadjuvant chemotherapy, N (%)			0.114
None	162 (63.0)	58 (52.3)	
Non-taxane	17 (6.6)	7 (6.3)	
Taxane	78 (30.4)	46 (41.4)	
Adjuvant chemotherapy, N (%)			0.779
None	84 (32.7)	38 (34.2)	
Non-taxane	32 (12.5)	11 (9.9)	
Taxane	141 (54.9)	62 (55.9)	
Radiotherapy, N (%)			0.901
Yes	147 (57.2)	62 (55.9)	
No	110 (42.8)	49 (44.1)	
Endocrine Therapy, N (%)			0.945
Yes	167 (65.0)	71 (64.0)	
No	90 (35.0)	40 (36.0)	
Draw Inject Same Side, N (%)			0.296
Yes	122 (47.5)	60 (54.1)	
No	135 (52.5)	51 (45.9)	
BP Same Side, N (%)			0.523
Yes	136 (52.9)	54 (48.6)	
No	121 (47.1)	57 (51.4)	
Complication, N (%)			0.128
None	179 (69.6)	84 (75.7)	
Hematoma	2 (0.8)	3 (2.7)	
Infection	25 (9.7)	11 (9.9)	
Seroma	51 (19.8)	13 (11.7)	
Tumor Recur, N (%)			0.107
Yes	7 (2.7)	0 (0.0)	
No	250 (97.3)	111 (100.0)	
Exercise Time, N (%)			0.413
<30min	134 (52.1)	52 (46.8)	
≥30min	123 (47.9)	59 (53.2)	
Exercise Freq, N (%)			0.827
<3/week	148 (57.6)	66 (59.5)	
≥3/week	109 (42.4)	45 (40.5)	
Exercise Type, N (%)			0.445
Jog	87 (33.9)	40 (36.0)	
Walk	84 (32.7)	29 (26.1)	
Rehab	86 (33.5)	42 (37.8)	
Healthy Diet, N (%)			0.874
Yes	128 (49.8)	57 (51.4)	
No	129 (50.2)	54 (48.6)	
Edu BCRL, N (%)			0.426
Yes	108 (42.0)	41 (36.9)	
No	149 (58.0)	70 (63.1)	
Avoid Heavy Lifting, N (%)			0.665
Yes	124 (48.2)	57 (51.4)	
No	133 (51.8)	54 (48.6)	
Skin Clean Dry, N (%)			0.306
Yes	137 (53.3)	52 (46.8)	
No	120 (46.7)	59 (53.2)	
Rest If Pain, N (%)			0.056
Yes	136 (52.9)	46 (41.4)	
No	121 (47.1)	65 (58.6)	

BMI, ​body mass index; ER, ​estrogen receptor; HER-2, ​human epidermal growth factor receptor-2; PR, ​progesterone receptor; LIQ, located in lower inner quadrant; LOQ, located in lower outer quadrant; UIQ, located in upper inner quadrant; UOQ, located in upper outer quadrant; BCS, breast-conserving surgery; ALND, ​axillary lymph node dissection; SLNB, ​sentinel lymph node biopsy; Draw Inject Same Side, whether blood draws/injections were performed on the surgical side; BP Same Side, whether blood pressure was measured on the surgical side; Skin Clean Dry, whether the skin is maintained clean and dry; Rest If Pain, whether appropriate rest is taken when experiencing pain.

### Identification of risk factors

**Figure 1:**
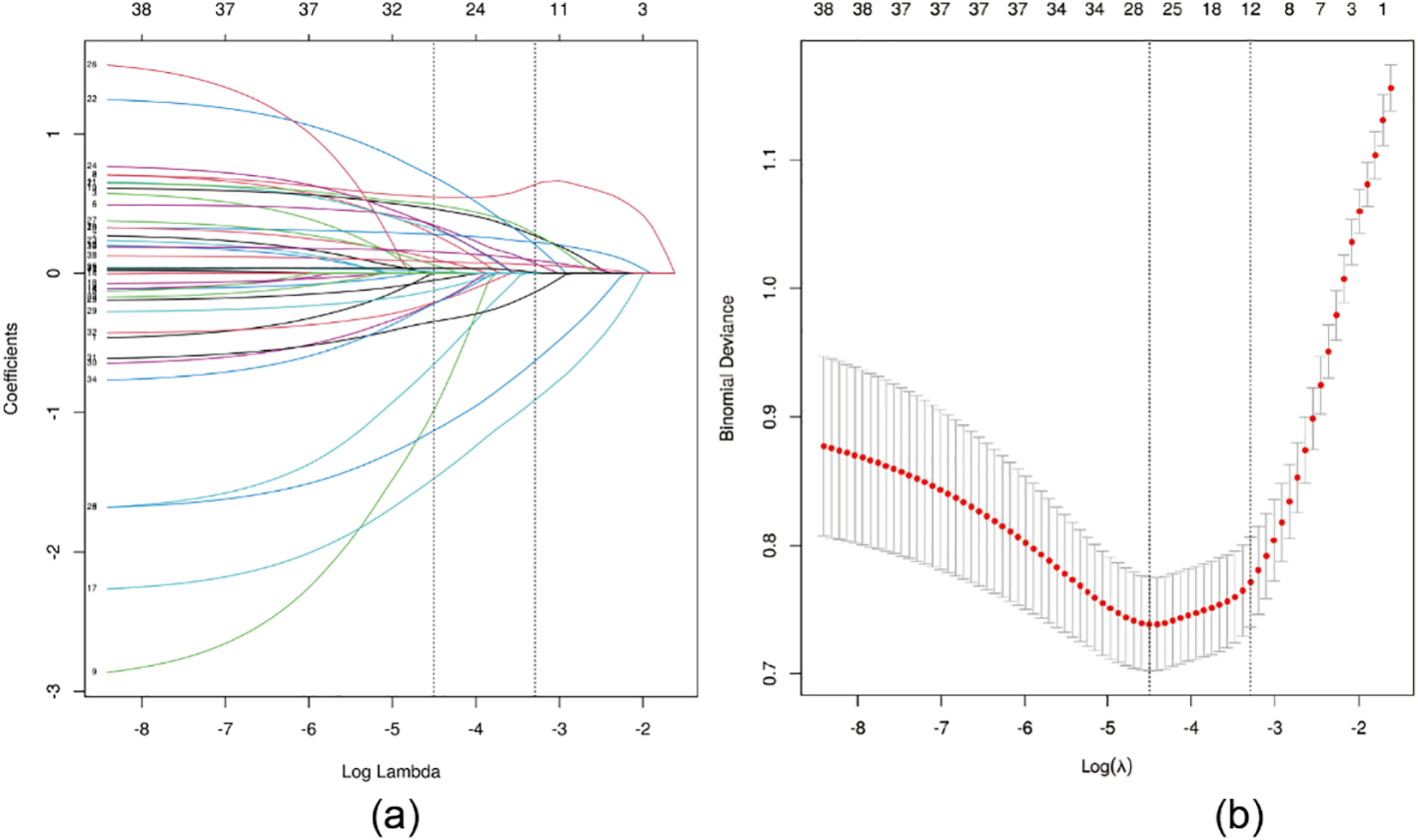
LASSO regression analysis was used to select characteristic factors. **(A)** The use of 10-fold cross-validation to draw vertical lines at selected values, where the optimal lambda produces 12 nonzero coefficients. **(B)** In the LASSO model, the coefficient profiles of 38 texture features were drawn from the log (λ) sequence. Vertical dotted lines are drawn at the minimum mean square error (λ = 0.011) and the standard error of the minimum distance (λ = 0.037).

LASSO regression was applied for feature selection, and optimal model parameters were determined via cross-validation to minimize overfitting and resolve multicollinearity issues (14). Based on the minimum lambda value of 0.037 within one standard error, 12 variables were selected from the initial 38 predictors. These included BMI, number of positive lymph nodes, number of dissected lymph nodes, clinical stage, N stage, lymph node dissection level, lymph node surgery type, neoadjuvant chemotherapy, postoperative radiotherapy, endocrine therapy, frequency of postoperative exercise, and BCRL-related health education (**Figure 1**).

### Comprehensive evaluation of model performance

**Figure 2:**
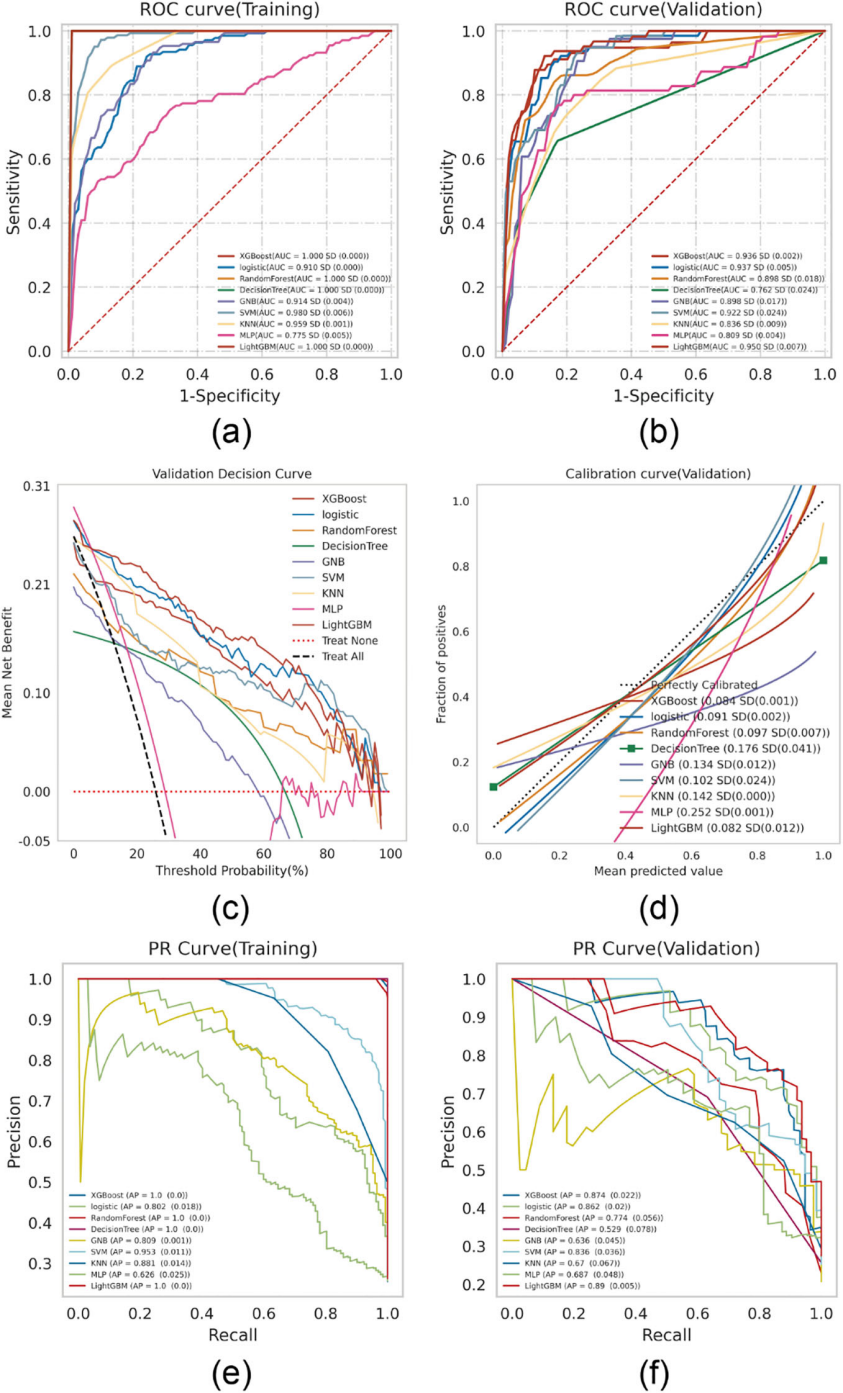
ML model comprehensive analysis. **(A)** Training sets ROC and AUC and **(B)** Testing sets ROC and AUC. Breast cancer patients were sampled 10 times at a ratio of 7:3. **(C)** Test set DCA where the black dotted line represents the assumption that all patients have BCRL and the red dotted line represents the assumption that no patient has BCRL. The remaining solid lines represent different models. **(D)** For the calibration curve of the test set, the abscissa is the average prediction probability, the case coordinate is the actual probability of the event, the dashed diagonal is the reference line, and the other smooth solid lines are the different model fitting lines. The closer the fitting line is to the reference line, the smaller the value in brackets is, the more accurate the model prediction value is. **(E)** Training set PR curve and AP and **(F)** testing set PR curve and AP. The y-axis is precision and the x-axis is recall. If the PR curve of one model is completely covered by the PR curve of another model, it can be concluded that the latter is better than the former, and the higher the AP value, the better the model performance. The different colors in the picture represent the corresponding model.

Nine machine learning algorithms—XGBoost, Logistic Regression, Random Forest, Decision Tree, Gaussian Naive Bayes (GNB), Support Vector Machine (SVM), k-Nearest Neighbor (KNN), Multilayer Perceptron (MLP), and LightGBM—were used to build predictive models on the training set and internally validated on the testing set. Grid search combined with 10-fold cross-validation was employed to optimize model performance. Model discrimination was primarily evaluated using the area under the curve (AUC) of the receiver operating characteristic (ROC) (15). Results showed that XGBoost, LightGBM, and Random Forest had the highest AUCs in the training set, while Logistic Regression and LightGBM performed best in the testing set (**Figures 2A, B**). Additional details on the performance metrics of all nine models are provided in **Supplementary Table 1**. The results indicate that complex ensemble models such as XGBoost, LightGBM, Random Forest, and Decision Tree achieved near-perfect performance in the training set (AUC: 1.00), but their performance decreased markedly in the validation set, with notable declines in accuracy and F1 scores, suggesting potential overfitting. In contrast, the Logistic Regression model demonstrated remarkable stability, with performance metrics remaining balanced and consistent between the training and validation sets, supporting its selection as the final optimized model. As AUC reflects predictive accuracy but not necessarily clinical utility (16), further assessments were conducted using decision curve analysis (DCA), calibration plots, and precision-recall (PR) curves.

DCA revealed that both Logistic Regression and XGBoost demonstrated superior clinical utility (**Figure 2C**). Calibration plots indicated that the predictive accuracy of XGBoost, Logistic Regression, and LightGBM was higher than other models (**Figure 2D**). Furthermore, a comprehensive analysis incorporating the above results and the average precision (AP) values from both the training and testing sets (**Figures 2E, F**) suggests that XGBoost and LightGBM are likely to exhibit overfitting. In contrast, logistic regression demonstrates relatively better stability and can be considered the optimal model.

### Construction and evaluation of the optimal model

**Figure 3:**
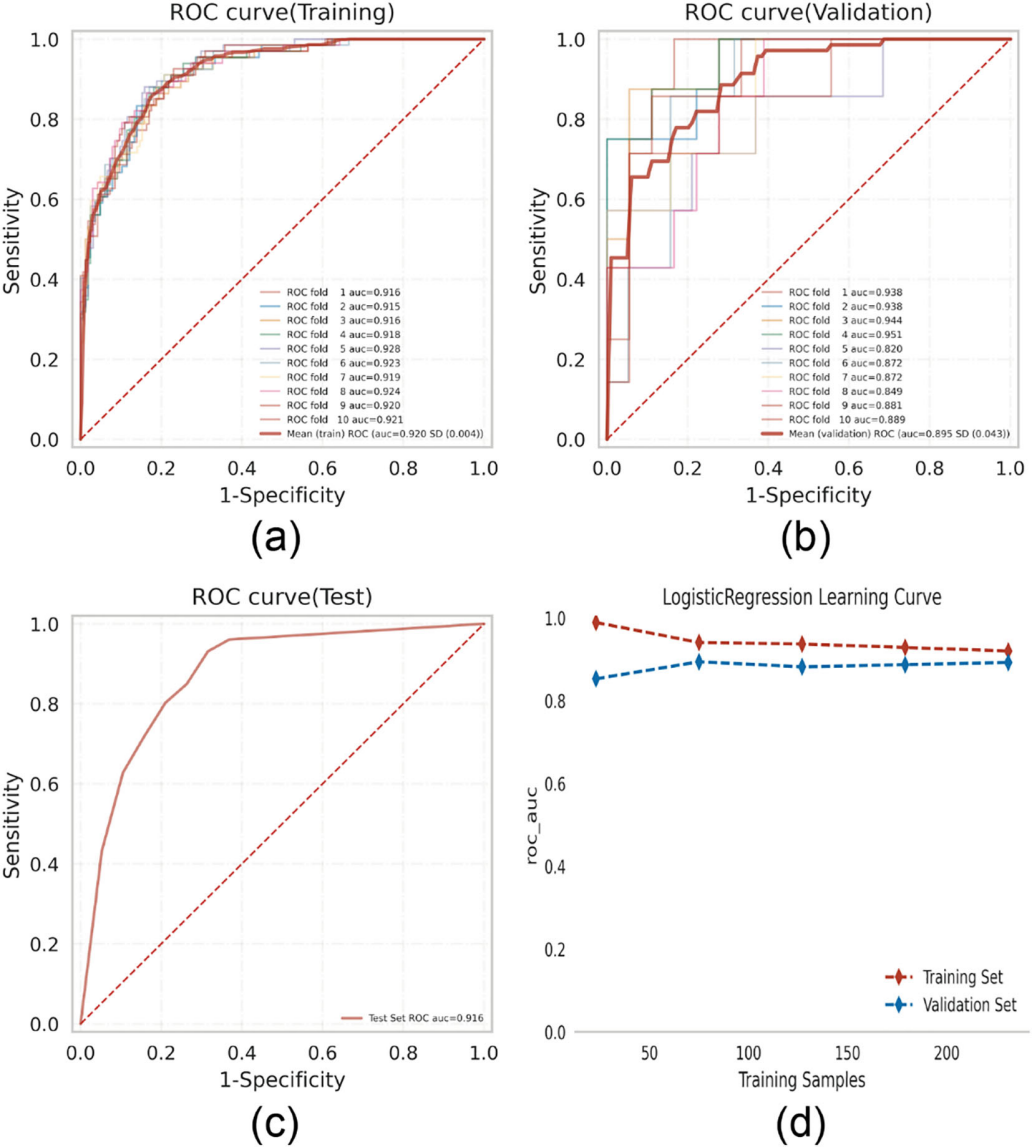
Logistic regression model training, validation, and testing. **(A)** Training sets ROC and AUC and **(B)** validation sets ROC and AUC. Training and cross-validation of 10% of breast cancer patients. Solid lines of different colors represent 10 different results. **(C)** Test set ROC and AUC. Test results for 30% of gout patients. **(D)** Learning curve. The red dashed line represents the training set and the blue dashed line represents the validation set.

Logistic regression analysis with 10-fold cross-validation was performed on the training set. The model achieved a mean AUC of 0.920 in the training set, 0.895 in the validation set, and 0.916 in the testing set (**Figures 3A–C**). The AUC values remained stable around 0.9 across all subsets, indicating high predictive accuracy. Given that the AUC in the validation set was not substantially lower than that of the testing set (within a 10% threshold), the model was considered well-fitted. Learning curves confirmed a good fit and high stability between the training and validation sets (17) (**Figure 3D**). These results support the suitability of the logistic regression model for classification tasks in this dataset.

### SHAP-based model interpretation

**Figure 4:**
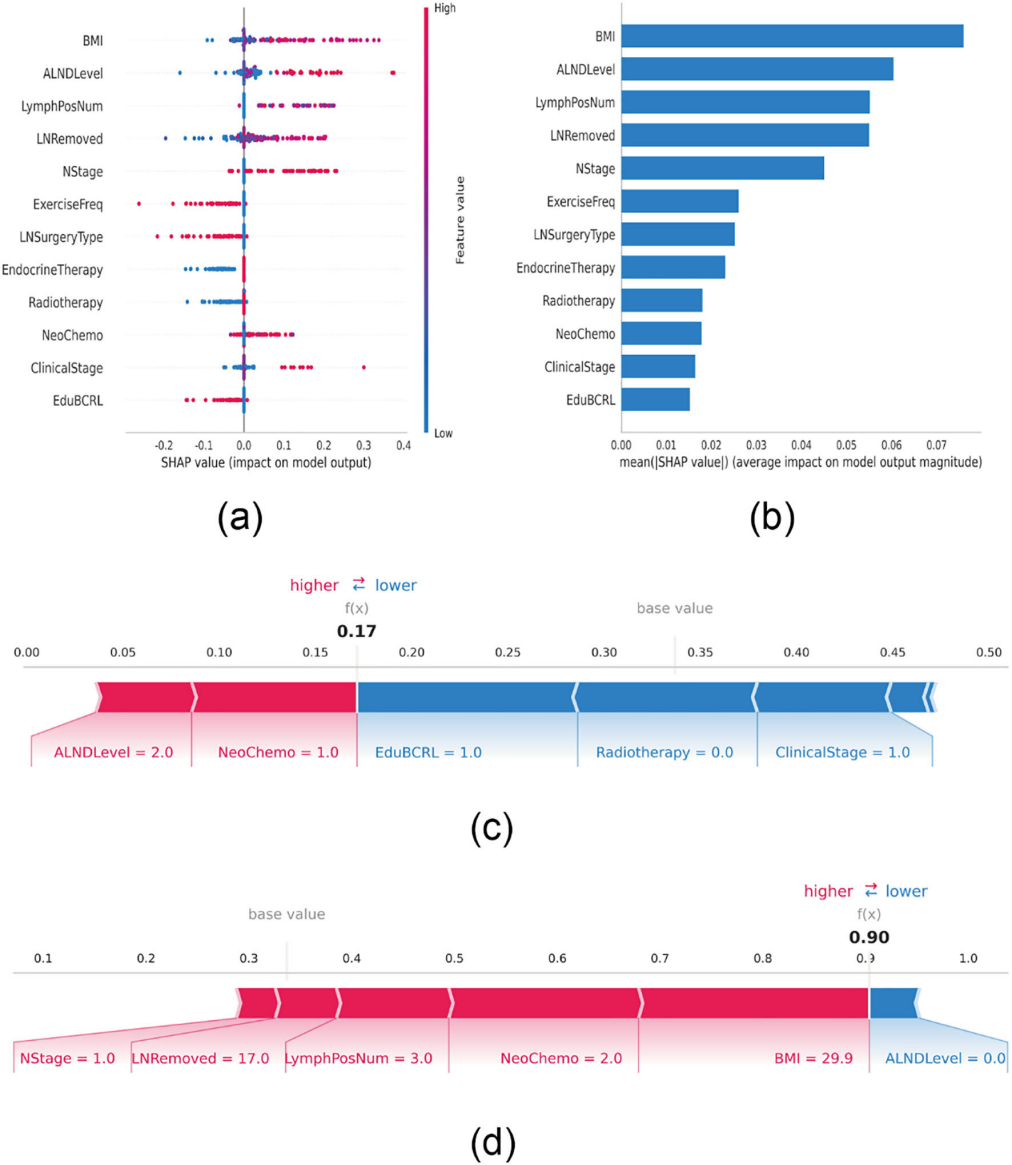
SHAP interprets the model. **(A)** Attributes of characteristics in SHAP. Each line represents a feature, and the abscissa is the SHAP value. Red dots represent higher eigenvalues and blue dots represent lower eigenvalues. **(B)** Feature importance ranking as indicated by SHAP. The matrix diagram describes the importance of each covariate in the development of the final prediction model. **(C)** Individual efforts by patients without BCRL and **(D)** with BCRL. The SHAP value represents the predictive characteristics of individual patients and the contribution of each to the predictive disease prevalence. The number in bold is the probability forecast value (f(x)), while the base value is the predicted value without providing input to the model. F(x) is the logarithmic ratio of each observation. Red features indicate increased risk of disease and blue features indicate reduced risk of disease. The length of the arrows helps visualize the extent to which the prediction is affected. The longer the arrow, the greater the effect.

To visually interpret the contributions of each selected variable, SHAP analysis was performed to illustrate how individual features influenced BCRL prediction within the model. As shown in **Figure 4A**, the top 12 predictive features were plotted, where each dot represents an individual patient’s contribution to the model output, with red indicating higher risk and blue indicating lower risk. Increased BMI, lymph node dissection level, number of positive lymph nodes, N stage, and clinical stage were all associated with elevated BCRL risk.

**Figure 4B** ranks the 12 risk factors based on their average absolute SHAP values, with the X-axis representing their relative importance in the model. It is important to note that SHAP values inherently represent the contribution of each feature to the model’s output. Their magnitude is relative rather than absolute, and features with larger absolute SHAP values indicate a stronger influence on the predicted outcome for a specific patient. To further demonstrate model interpretability, two representative patient cases are presented. One patient without BCRL had a low SHAP prediction score of 0.17, characterized by multiple protective features, including participation in BCRL-related health education, absence of radiotherapy, and an early clinical stage (Stage I). These factors collectively lowered the predicted risk from the baseline value, with the lengths of the blue bars reflecting the “pulling effect” of each protective factor in reducing risk (**Figure 4C**). In contrast, another patient diagnosed with BCRL had a high score of 0.90, exhibiting several risk-enhancing features, including a high BMI (29.9 kg/m²), regional lymph node metastasis (N1), extensive lymph node dissection, three positive lymph nodes, and neoadjuvant chemotherapy with a taxane regimen. These factors substantially increased the predicted probability, with the lengths of the red bars visually indicating the “pushing effect” of each risk factor in elevating disease risk (**Figure 4D**).

Furthermore, to present a comprehensive clinical decision support workflow, we translated our machine learning model into actionable clinical practice (**Supplementary Figure S1**). The framework begins with comprehensive data collection encompassing 38 variables, identifies 12 key predictive factors via LASSO regression to optimize model efficiency, and integrates SHAP-based explanations to support individualized risk profiling. This provides a systematic strategy for implementing personalized BCRL prevention in routine breast cancer care.

## Discussion

The primary objective of this study is to develop a predictive model for breast cancer-related lymphedema (BCRL) using machine learning algorithms. Twelve clinical variables were selected from an initial set of 38 using LASSO regression to assess the risk of lymphedema in postoperative breast cancer patients. Multiple machine learning models were subsequently implemented and optimized. After comparing performance across AUC, DCA, calibration curves, and PR curves, the logistic regression model demonstrated superior performance compared to other ML models. Furthermore, SHAP values were employed for model interpretability, identifying several key predictors of BCRL. Among them, BMI, axillary lymph node dissection (ALND), and lymph node status showed the highest SHAP values, indicating a greater influence on model prediction and highlighting their clinical relevance.

These findings are consistent with prior research. Multiple retrospective cohort studies have identified BMI as the most significant patient-related risk factor for BCRL and emphasized weight reduction as a preventive strategy (12, 18). Additionally, another study demonstrated that preoperative BMI influences both the incidence and timing of BCRL. Once BCRL occurs, patients with higher BMI tend to experience slower recovery, with lymphedema persisting in 50.0% of cases after 5 years (19).Mechanistically, obesity has been found to negatively affect lymphatic vessel density, endothelial cell proliferation, lymphatic drainage, collecting vessel pumping efficiency, and macromolecular clearance, all of which contribute to impaired lymphatic absorption (20, 21).

In this study, the second most significant risk factor is the level of axillary lymph node dissection. As a surgical procedure that disrupts the lymphatic network, ALND remains strongly associated with BCRL even after adjusting for the number of dissected lymph nodes (22). Despite advances in early diagnosis and treatment of breast cancer, ALND still results in upper limb lymphedema in 30–50% of patients. This condition is characterized by the abnormal accumulation of protein-rich interstitial fluid, leading to swelling and tissue remodeling (23). The use of breast lymphatic level (BLL)-based ALND, involving stepwise excision of lymph nodes in patients with positive axillary involvement, has been shown to reduce surgical extent while preserving oncological outcomes and potentially lowering BCRL incidence (24).

The influence of lymph node status on the development of BCRL remains a topic of ongoing debate. Our findings indicate that both the number of positive lymph nodes and the number of nodes removed during surgery are important risk factors. According to Huang et al., axillary lymph node status significantly impacts treatment and prognosis in breast cancer, likely due to broader dissection ranges, increased numbers of positive nodes, and the use of radiotherapy or chemotherapy (25). These observations are consistent with our results. A high number of positive lymph nodes (>8) may serve as a surrogate marker for extensive lymphatic metastasis and treatment-related injury, often necessitating more aggressive ALND, which increases the risk of BCRL (26, 27). Multivariate analysis conducted by Kwan et al. further supports the prognostic value of pathological lymph node counts in predicting BCRL severity. These identifiable risk factors enable reliable prediction of BCRL and support more tailored treatment planning by clinicians (6, 28).

Additionally, this study is the first to incorporate postoperative health behavior factors—such as rehabilitation exercises, limb management, and lifestyle interventions—into model development, revealing their potential protective effects. Behavioral factors such as “not ignoring upper limb swelling,” “avoiding strenuous activity of the affected limb,” “avoiding lifting heavy objects with the affected limb,” and “avoiding fatigue of the affected limb” are significantly associated with reduced BCRL risk. This may be due to excessive use of the affected limb causing increased muscle tension, which impairs lymphatic return and triggers lymphedema (29). Previous studies have shown that early, structured rehabilitative exercise enhances blood and lymphatic circulation, reducing interstitial lymph accumulation and the severity of lymphedema (30).The current study further verifies these findings using quantitative machine learning models.

In this study, we observed a cumulative incidence of BCRL of 26.63%, which aligns closely with reports from multiple high-quality studies. For example, a systematic review and meta-analysis including 21 studies on BCRL reported a wide range of incidence rates from 6.4% to 76.3% (7). Recent large-scale cohort studies, such as those by Li et al. (31), Wu et al. (32) and Martínez-Jaimez et al. (18), reported pooled incidence rates of approximately 25%–30%, which are highly consistent with our findings. However, some studies focusing on modern surgical techniques, such as the widespread adoption of SLNB, reported lower incidence rates (6.4%–12.5%) (33). The relatively higher incidence in our cohort can be reasonably explained by several key factors. First, the surgical population composition: a considerable proportion of patients in our cohort underwent ALND, which remains a major risk factor for BCRL. Although SLNB has become the standard procedure for early-stage patients, ALND remains the routine approach for patients with clinically positive lymph nodes or metastatic sentinel nodes (34). The proportion of ALND in our study is consistent with other studies focusing on populations requiring this procedure. Second, the sensitivity of diagnostic criteria: we used arm circumference measurement (≥2.0 cm difference) as the diagnostic criterion for BCRL, a method widely used in clinical practice due to its simplicity and good clinical relevance. Compared with stricter objective methods, such as bioimpedance spectroscopy (BIS) or measurements using <2.0 cm thresholds, this method is more sensitive and may capture mild to moderate cases (35). Third, the impact of radiotherapy: a relatively high proportion of patients in our cohort received adjuvant radiotherapy, particularly targeting the axillary or supraclavicular regions. Regional lymph node irradiation is known to significantly increase the risk of lymphatic injury and fibrosis, acting synergistically with surgery to elevate BCRL risk (36). Finally, BCRL is a late-onset complication, with risk persisting for several years postoperatively. The relatively long follow-up period in our study ensured adequate capture of late-onset cases, which may be underestimated in studies with shorter follow-up durations. Taken together, the 26.63% incidence reported in our study accurately reflects the disease burden of BCRL in a modern cohort that includes patients at intermediate to high risk (e.g., those undergoing ALND and radiotherapy), employs sensitive clinical diagnostic criteria, and has been followed longitudinally. This comparison underscores the importance of considering differences in patient populations, treatment modalities, diagnostic criteria, and follow-up strategies when interpreting and comparing BCRL incidence across studies.

## Strengths and limitations of the study

Compared with traditional statistical approaches, machine learning models demonstrated superior predictive performance in this study. Among nine tested models, logistic regression showed the best balance between predictive accuracy and classification ability, likely due to the strict inclusion criteria that minimized model noise. Notably, SHAP analysis allowed for the quantification of each predictor’s contribution, enabling the model to be not only predictive but also interpretable. This transparency is essential for clinical risk assessment and facilitates the development of individualized follow-up and intervention plans.

Nevertheless, several limitations should be acknowledged. First, as a retrospective study, selection bias may exist. Second, the dataset was derived from a single center with a relatively small sample size, which may limit generalizability. Third, some variables—such as frequency of limb usage and subjective sensory scores—were based on patient records and follow-up interviews and may be prone to measurement bias. Lastly, the model did not include imaging indicators such as lymphangiography or elastography, which could provide additional objective data and further improve predictive accuracy and clinical relevance in future research.

To address the concern regarding the generalizability of findings derived from a single-center cohort, we compared the main demographic and clinical characteristics of our study population with those reported in large national and international breast cancer cohorts. The mean BMI values in the Non-lymphedema group (24.24 kg/m²) and the Lymphedema group (25.96 kg/m²) in our study were comparable to those reported in two large prospective Chinese cohorts (37, 38) and a multicenter case–control study conducted in the United States (39). In addition, the prevalence of axillary lymph node dissection (ALND) in our cohort was consistent with the rates reported in a recent systematic review and meta-analysis of regional therapies for breast cancer (40), reflecting contemporary surgical practice in which ALND remains applied to selected patients despite the increasing adoption of sentinel lymph node biopsy (SLNB). Meanwhile, the distribution of clinical stages and the proportion of patients receiving adjuvant chemotherapy or radiotherapy were also in line with the characteristics described in a recent retrospective study of breast cancer populations (12). Collectively, these comparisons indicate that although our study was conducted within a single institution, the sample shares similar demographic and clinical features with broader breast cancer populations, supporting the potential external validity and generalizability of our predictive model.

Therefore, future studies should aim to conduct multi-center, prospective cohort investigations and integrate multi-dimensional data, including imaging, genomics, and behavioral monitoring. Embedding such predictive models into clinical follow-up systems to enable dynamic postoperative risk evaluation and personalized alerts will be critical for real-world implementation.

## Conclusion

In this study, a machine learning–based individualized prediction model for postoperative lymphedema in breast cancer patients has been successfully developed, demonstrating robust predictive performance and promising clinical applicability. Additionally, SHAP-based interpretation facilitates personalized risk assessment for BCRL among breast cancer patients. This effective, computer-assisted approach may serve as an important tool for precision rehabilitation management and represents a shift from empirical prevention strategies toward data-driven clinical decision-making.

## Data Availability

The raw data supporting the conclusions of this article will be made available by the authors, without undue reservation.

## Supplementary material

Supplementary Figure 1 Clinical decision support workflow for breast cancer-related lymphedema risk assessment.Comprehensive five-stage framework for implementing machine learning-based BCRL risk prediction in clinical practice: (1) Patient Assessment: multimodal data collection; (2) Feature Extraction: automated processing of 12 validated predictors; (3) Model Prediction: individualized risk computation with SHAP interpretability; (4) Risk Interpretation: visualization of feature contributions and modifiable factors; (5) Clinical Action: personalized prevention strategies based on identified risk profile. This workflow bridges predictive analytics with clinical decision-making to enable precision prevention of BCRL.
