# Supplementary figures and images for: Machine learning-based personalized risk prediction model for breast cancer-related lymphedema after surgery
Source: Front Oncol. 2025 Dec 4;15:1729340. doi: 10.3389/fonc.2025.1729340 (PMC12711468; doi:10.3389/fonc.2025.1729340)

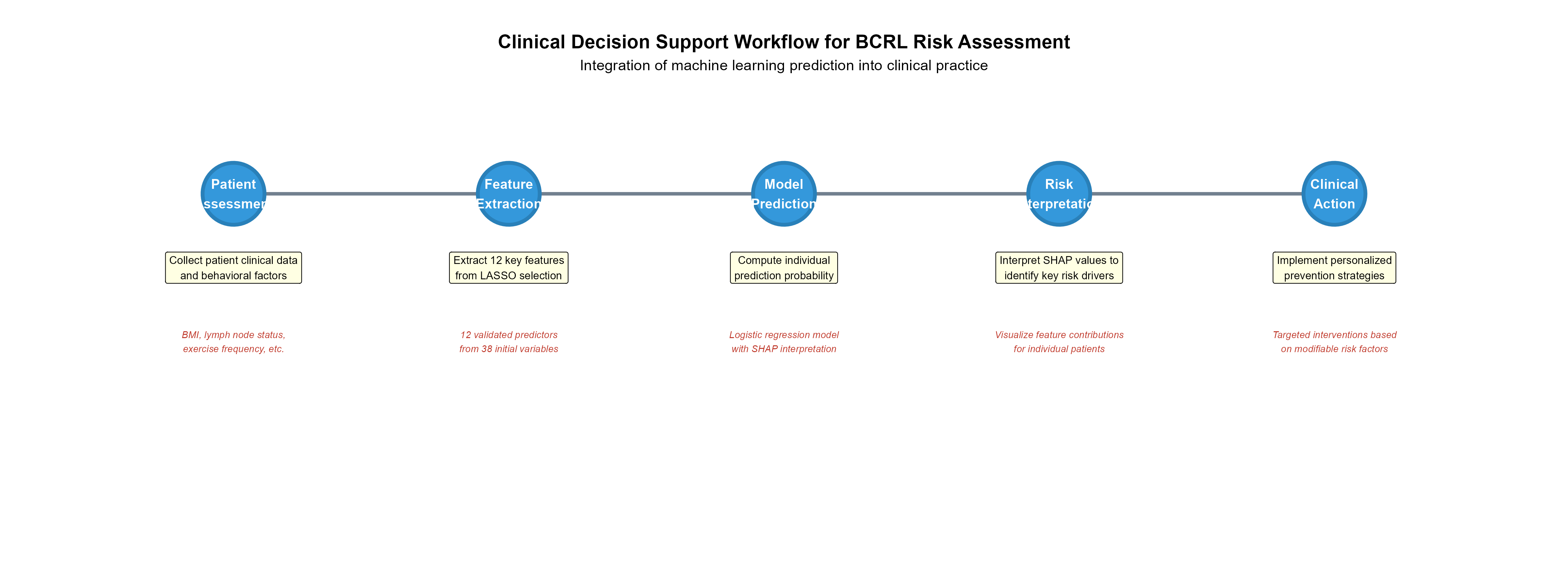

Supplement: Supplementary Figure 1 — Clinical decision support workflow for breast cancer-related lymphedema risk assessment.Comprehensive five-stage framework for implementing machine learning-based BCRL risk prediction in clinical practice: (1) Patient Assessment: multimodal data collection; (2) Feature Extraction: automated processing of 12 validated predictors; (3) Model Prediction: individualized risk computation with SHAP interpretability; (4) Risk Interpretation: visualization of feature contributions and modifiable factors; (5) Clinical Action: personalized prevention strategies based on identified risk profile. This workflow bridges predictive analytics with clinical decision-making to enable precision prevention of BCRL. [file Image1.png]
